# Expanding global access to essential medicines: investment priorities for sustainably strengthening medical product regulatory systems

**DOI:** 10.1186/s12992-018-0421-2

**Published:** 2018-11-01

**Authors:** Lukas Roth, Daniel Bempong, Joseph B. Babigumira, Shabir Banoo, Emer Cooke, David Jeffreys, Lombe Kasonde, Hubert G. M. Leufkens, John C. W. Lim, Murray Lumpkin, Gugu Mahlangu, Rosanna W. Peeling, Helen Rees, Margareth Ndomondo-Sigonda, Andy Stergachis, Mike Ward, Jude Nwokike

**Affiliations:** 1 United States Pharmacopeia, Rockville, USA; 2 Global Medicines Program, School of Pharmacy, School of Public Health, University of Washington, Seattle, USA; 3 Right to Care, South African Health Products Regulatory Authority, Cape Town, South Africa; 4 World Health Organization, Geneva, Switzerland; 5 International Federation of Pharmaceutical Manufacturers and Associations Regulatory Science Committee, Geneva, Switzerland; 6 World Bank, Washington, USA; 7 Division of Pharmacoepidemiology and Clinical Pharmacology, University of Utrecht, Utrecht, Netherlands; 8 Centre of Regulatory Excellence, Duke-National University of Singapore Medical School, Singapore, Singapore; 9 Bill and Melinda Gates Foundation, Seattle, USA; 10 Medicines Control Authority Zimbabwe, Harare, Zimbabwe; 11 London School of Hygiene and Tropical Medicine, London, UK; 12 South African Health Products Regulatory Authority, University of Witwatersand, Johannesburg, South Africa; 13 New Partnership for Africa’s Development, Midrand, South Africa; 14 Global Medicines Program, School of Pharmacy, School of Public Health, University of Washington, Seattle, USA; 15 Promoting the Quality of Medicines program, United States Pharmacopeia, Rockville, USA

**Keywords:** Access to essential medicines, Substandard and falsified, Regulatory system strengthening

## Abstract

Access to quality-assured medical products improves health and save lives. However, one third of the world’s population lacks timely access to quality-assured medicines while estimates indicate that at least 10% of medicine in low- and middle-income countries (LMICs) are substandard or falsified (SF), costing approximately US$ 31 billion annually. National regulatory authorities are the key government institutions that promote access to quality-assured medicines and combat SF medical products but despite progress, regulatory capacity in LMICs is still insufficient. Continued and increased investment in regulatory system strengthening (RSS) is needed. We have therefore reviewed existing global normative documents and resources and engaged with our networks of global partners and stakeholders to identify three critical challenges being faced by NRAs in LMICs that are limiting access to medical products and impeding detection of and response to SF medicines. The challenges are; implementing value-added regulatory practices that best utilize available resources, a lack of timely access to new, quality medical products, and limited evidence-based data to support post-marketing regulatory actions. To address these challenges, we have identified seven focused strategies; advancing and leveraging convergence and reliance initiatives, institutionalizing sustainability, utilizing risk-based approaches for resource allocation, strengthening registration efficiency and timeliness, strengthening inspection capacity and effectiveness, developing and implementing risk-based post-marketing quality surveillance systems, and strengthening regulatory management of manufacturing variations. These proposed solutions are underpinned by 13 focused recommendations, which we believe, if financed, technically supported and implemented, will lead to stronger health system and as a consequence, positive health outcomes.

## Background

Access^1^ to quality-assured medical products^2^ improves health and saves lives. Nonetheless, according to the World Health Organization (WHO), one-third of the world’s population lacks timely access to quality assured medicines [2]. Compounding this is the growing global concern of substandard and falsified (SF) medical products, which WHO estimates constitute at least 10% of medicines in low- and middle-income countries (LMICs) and costs these countries US$ 31 billion annually [3]. Their prevalence jeopardizes patient safety, diminishes confidence in health systems, increases treatment failure, wastes resources, and contributes to antimicrobial resistance [3, 4]. National regulatory authorities (NRAs) are the key government institutions that promote access to quality products and combat SF medicines, as called for by World Health Assembly (WHA) Resolution 67.20 on regulatory system strengthening (RSS) for medical products [5].

Considerable progress has been made to strengthen medical product regulatory systems but capacity in many LMICs is still insufficient and indeed, sometimes a barrier to access to medicines [6–9]. Increased investments in RSS are therefore paramount to the attainment of mature regulatory systems. Recent global normative documents have identified RSS priorities [5–7, 10, 11]. This paper builds upon these documents and further defines the key RSS investment priorities needed to ensure timely access to new medical products and to protect against SF medical products. To accomplish this, we identify three critical challenges LMICs currently face: –implementing value-added regulatory practices that best utilize available resources; a lack of timely access to new, quality medical products; and limited evidence-based data to support post-marketing regulatory actions. We also propose strategies and 13 focused recommendations (Table 1) for where investments in regulatory systems are needed over the next decade to achieve efficiencies, promote public confidence in health systems, and ensure maximum public health impact.

**Table 1:** Summary of challenges, proposed strategies, and recommendations

Challenge	Proposed Strategy	No.	Recommendation	Recommendation Type^a^
Implement value-added regulatory practices that utilize available resources	Advance and leverage convergence and reliance initiatives	1	Document and communicate current reliance and convergence efforts and develop supporting infrastructure and tools to facilitate implementation	Analytics
		2	Strengthen capacity building networks	Collaboration
	Institutionalize sustainability	3	Define needed capacities in NRAs using the WHO global benchmarking tool	Analytics
		4	Establish stable and transparent financing mechanisms	System development
	Utilize risk-based approaches for resource allocation	5	Perform risk analysis and implement risk management	Workforce development
		6	Develop systems to monitor and evaluate the impact of risk-based approaches for resource allocation	Analytics
Timely access to new quality-assured medical products without compromising safety and efficacy	Strengthen registration efficiency and timeliness	7	Establish and refine value-added registration processes, resources, and systems	System development Collaboration
		8	Build value-added technical capacity of assessors	Workforce development
	Strengthen inspection capacity and effectiveness	9	Enhance information sharing and use and reliance on existing inspection resources	Collaboration
		10	Build capacity of multi-disciplinary teams of inspectors	Workforce development
Limited evidence-based data to support post-marketing regulatory action	Develop and implement risk-based post-marketing quality surveillance systems	11	Establish recognition of the value for risk-based post-marketing quality surveillance throughout the supply chain	System development
		12	Develop and implement risk-based post-marketing quality surveillance programs, supporting tools, and communication strategies	Analytics Workforce development
	Strengthen regulatory management of manufacturing variations	13	Develop and implement risk-based programs to incorporate post-marketing manufacturing variations into marketing authorizations	Analytics System development

^a^These recommendation types were defined by the authors to classify how the recommendations might be implemented:

Analytics – Generating and interpreting data collected through the implementation of activities and general research

Collaboration – Coordinating and communicating within and among NRAs, their stakeholders and other technical partners

System development – Establishing processes, procedures and platforms to enhance and facilitate activities

Workforce development – Building the capacity of staff

## Methods

The five normative documents [5–7, 10, 11] were identified by the authors through discussions with and recommendations from colleagues and partners working in this field and our own collective experience and expertise. These sources, while not necessarily exhaustive, represent a consolidated list of well-researched seminal pieces published by various global health organizations and global health experts within the last 10 years, which center overarchingly on health system strengthening and access to medicines and identify important and novel ideas and suggestions. We have sought to build upon these existing resources by taking existing best practices, relevant ideas and suggestions and providing an additional level of detail and identifying entirely new recommendations through our experiences and the review of additional, related literature. The challenges, strategies and recommendations selected were those we felt were the highest priority and most likely to lead to sustainable positive change to global regulatory systems.

The information in Table 2 was collected through informal discussions with NRA staff, consultants, and other technical experts.

**Table 2:** Financing mechanism for selected NRAs^a^

Country (NRA)	Structure	Funding Source(s)	Comments
Argentina (Administración Nacional de Medicamentos Alimentos y Tecnología)	Autonomous	Government funds	Administratively and financially independent but user fees go to central funding; decision-making is independent.
Australia (Therapeutic Goods Administration)	Autonomous	User fees	Regulatory decisions are made by delegates of the Ministry of Health (TGA employees)
Ethiopia (Food and Medicine and Health Care Administration and Control Authority)	Semi-autonomous	Government funds User fees	Under Department of Health but reports to Parliament
Ghana (Food and Drugs Authority)	Operationally autonomous (not financially)	Government funds User fees	Does not sit under the Ministry of Health; independent agency that reports to the Minister
India (Drug Controller General of India, Drug Control Authority)	Semi-autonomous, under Ministry of Health	Government funds	Minimal user fees, which are unsustainable and provided to the Ministry
Indonesia (Badan Pengawas Obat dan Makanan)	Ministry level institution	Government funds	Minimal user fees; head of BPOM is a minister-level position, reports to President
Lao People’s Democratic Republic (Food and Drug Department)	Not autonomous, under Ministry of Health	Government funds Donors	
Netherlands (Medicines Evaluation Board)	Autonomous	User fees	Regulatory decisions are independent of Ministry
Pakistan (Drug Regulatory Authority Pakistan)	Autonomous	Government funds User fees	Under Ministry of Health but independent in its decision-making; minimal government funding (~ 2%)
Papua New Guinea (Pharmaceutical Service Standards Branch)	Not autonomous, under Department of Health	Government funds	Fees are returned to Treasury
Singapore (Health Sciences Authority^a^)	Autonomous, under Ministry of Health	Government funds User fees	Statutory board under Ministry of Health, autonomy in decision-making
South Africa (South African Health Products Regulatory Authority)	Autonomous, under the Department of Health	Government funds User fees	Independent public entity that retains revenue generated, employs its own staff, and is accountable to Parliament
United States of America (Food and Drug Administration)	Autonomous, under Department of Health and Human Services	Government funds User fees	Fee proportions varies by centers; decisions made almost exclusively by civil servants in FDA (delegated decision-making by law and regulation)
Zimbabwe (Medicines Control Authority of Zimbabwe)	Autonomous, not under Ministry of Health	User fees	Not under Ministry of Health, but Minister is responsible for actions

^a^Health Products Regulation Group

### Implement value-added regulatory practices that utilize available resources

#### Advance and leverage convergence and reliance initiatives

**Fig. 1:**
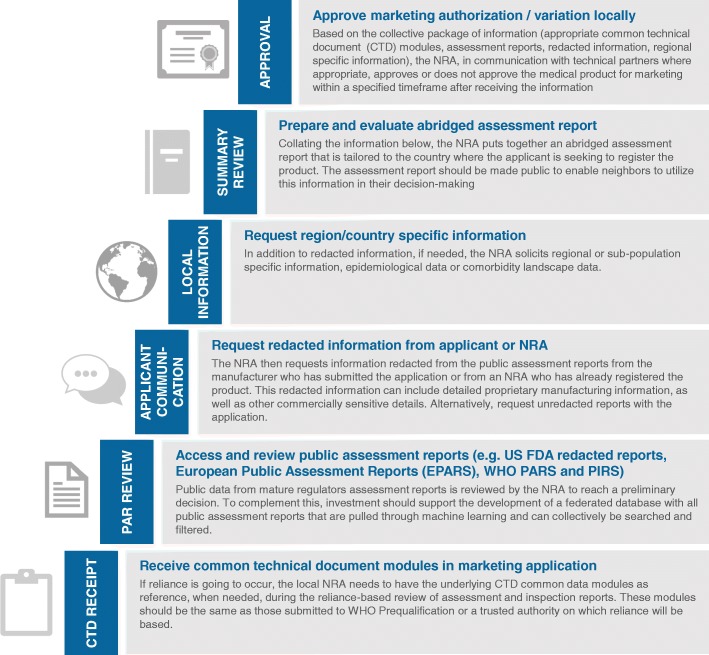
Operationalizing regulatory reliance for registration of medical products

As the globalization of pharmaceutical manufacturing continues, NRAs struggle to individually regulate existing and new medical products [11]. An effective way to tackle this challenge is to continue advancing the adoption of workable regulatory reliance models, while leveraging complementary convergence efforts. Regulatory reliance refers to a sovereign authority using the work products of trusted authorities and organizations to inform a regulatory decision based on local settings and their own scientific knowledge, knowledge of the local health care system and culture, and regulatory procedures (see Fig. 1) [12, 13]. Regulatory convergence, complementarily, entails country regulatory processes and technical requirements becoming more aligned over time [14]. Implementation of reliance initiatives will differ by regulatory functions (e.g., registration vs. pharmacovigilance) and will need to be tailored so should take into account available resources and the previous work and experience of other NRAs [15, 16].

#### Document and communicate current reliance and convergence efforts and develop supporting infrastructure and tools to facilitate implementation

Investment is needed to further document and communicate the status of current reliance and convergence efforts. These efforts are occurring in many regions, e.g., − the East African Community, the Zazibona initiative (a collaboration among several countries of the Southern African Development Community), and the Caribbean Community [6, 12, 17]. However, the work of these types of initiatives is often poorly documented and communicated. We believe the broader global health community needs to be better informed about these efforts. One mechanism for improved communication is to develop a public database hosted by WHO and populated by regulators and their technical partners. Existing reliance and convergence efforts should be identified, and data on who is leading the work, where it is taking place, the intended objectives, and current status should be listed. This should be an integral part of WHO’s Coalition of Interested Partners (CIP), an initiative spearheaded by WHO seeking to achieve better coordination, efficiency and outcomes in regulatory system strengthening activities, and Good Reliance Practices initiatives [18]. It would enable stakeholders and other regions to better determine how they might engage in these activities, adapt their approaches based on existing best practices, and avoid duplicating efforts. Complementing this external collaboration is the need for a clear definition of the roles and responsibilities of all departments and institutions that form the regulatory system within a given country to avoid ambiguity and unnecessary complexity. In addition, we recommend the development of infrastructure and systems to continue operationalizing reliance as a twenty-first century regulatory best practice [11, 12, 19]. For example, a simple, modular, open-source platform that allows collation and simultaneous review of public assessment reports and confidential data sharing should be available. Its architecture should be adaptable, and administration of the platform should be simple and preserve the integrity of needed confidentiality and the decision-making process of NRAs.

#### Strengthen capacity building networks

Workforce development, along with developing a pipeline for the next generation of regulatory scientists, continues to be an invaluable factor in advancing value-added regulatory processes, including reliance efforts. Historically, the focus of many training efforts was based on how well-resourced NRAs approach their responsibilities, but such approaches are often inappropriate and impractical in low-resource settings. Curricula that focus on the specific capacities needed by LMIC regulators are lacking (e.g., oversight of local manufacturing, regulation through reliance and networks, local post-marketing activities, and management of regulatory administrative functions). Inconsistent training quality and uncoordinated training initiatives also limit the technical capacity of different regulators. WHO is working to address this by developing a global curriculum framework that will complement its global benchmarking tool (GBT) and define the requisite competencies for regulatory staff working across the different regulatory functions [20]. Investment is needed to (1) strengthen partnerships, such as WHO’s CIP [18], that are working to coordinate and ensure greater consistency and appropriate focus of capacity building efforts, and (2) strengthen training and technical assistance providers associated with networks such as the International Pharmaceutical Regulators Programme, the International Council for Harmonization’s Training Subcommittee, Asia-Pacific Economic Cooperation’s (APEC) Centers of Excellence, select academic institutions and leading regulatory agencies such as the African Medicines Regulatory Harmonization Initiative’s Regional Centres of Regulatory Excellence [17]. WHO and these institutions grasp the specific technical needs and regulatory goals of the NRAs they support and, when working together, can deliver appropriately focused, competency-based capacity building programs in a sustainable, coordinated, and effective manner.

### Institutionalize sustainability

NRAs are generally funded through a mix of taxes, user fees, and occasionally donors, such as the Global Fund to Fight AIDS, Tuberculosis and Malaria or the United States Agency for International Development Table 2 provides information on the financing mechanism of 14 illustrative NRAs that represent varying regulatory maturity levels, income classifications, and geographic regions. Many LMICs cannot adequately fund all of their public health needs and their NRAs are particularly vulnerable [17, 21, 22]. Defining clear goals and institutionalizing sustainability in terms of human and financial resources is therefore essential for effective long-term NRA functioning [7]. Sustainability ensures that the necessary processes and resources exist to enable the NRA to fulfill its mandate while remaining responsive, value-added, outcome-oriented, science-based, accountable, risk-proportionate, and independent [11]. To ensure this, any external investments should focus on supporting the development of infrastructure, strategies, systems, and staff rather than directly funding NRA operational activities.

## Define needed capacities in NRAs using the WHO global benchmarking tool

**Fig. 2:**
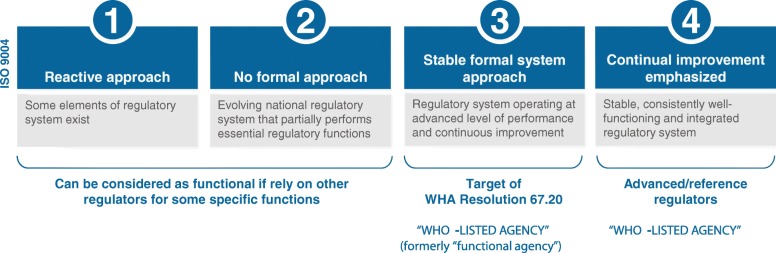
WHO global benchmarking tool maturity levels. Adapted with permission from the WHO NRA Regulatory System Strengthening Database [24]

As part of a continuous improvement process and in line with WHA Resolution 67.20, WHO has begun beta-testing and finalizing its harmonized medicines-vaccines GBT. The GBT uses an agreed-upon set of standards, indicators, and metrics that cover the enabling regulatory system and all of the major regulatory function modules. These standards have been published by WHO as factsheets, which act as rubrics to allow NRAs, WHO, and selected technical partners to assess an NRA’s maturity level (see Fig. 2) [23]. WHO and other technical partners have performed formal benchmarking of 17 countries and 39 additional countries have performed self-assessments [24]; 28 LMICs remain to be benchmarked. Once completed, these assessments need to be used to set realistic goals, identify barriers for NRAs to meet their regulatory functioning goals, define specific investment needs, and determine future capacity needs as more complex products and supply chains come to their markets. Plans to publicize NRAs reaching higher maturity levels as “WHO listed-authorities” (currently stringent regulatory authorities) [25] following GBT assessment by WHO, will also help promote trust, reliance, and transparency.

## Establish stable and transparent financing mechanisms

NRAs must ensure they have the legal mandate, political will, and related structures to properly finance operations, specifically through revenue generation and retention. The generation and retention of revenue means that an NRA requires less direct government funding and strengthens functional efficiency and accountability (Table 3). While many NRAs in LMICs levy fees, they often charge arbitrary amounts that do not cover their value-added activities. This can create barriers to both market entry and adequate post-marketing quality surveillance, circumvent reliance efforts, and hinder potential financial sustainability [11, 26, 27]. The African Union (AU) Model Law on Medical Product Regulation (Model Law) provides a reference guide to assist countries to review or enact laws with powers to levy, collect, and utilize fees for rendered services [28]. Once established, the legal mandate needs to be supported by the development of an appropriate, transparent and process-oriented fee structure commensurate with the regulatory workload required. These efforts should also be accompanied by support for the development of an institutional development plan that is based on the GBT assessment and provides a blueprint for an NRA to reach and maintain its desired maturity level and to establish systems that ensure efficient and risk-based management of human, financial and information technology resources [4, 23].

**Table 3:** The Medicines Control Authority of Zimbabwe (MCAZ) – An example of sustainable, risk-based regulation

*Due to a policy shift, in 1997, several Zimbabwean government entities, including MCAZ, were given the ability to run their operations by utilizing revenue generated through the provision of services. Since this change, MCAZ has performed several financial analyses and exercises to balance the need to cover the cost of service provision and the need to enable market entry for both domestic and international manufacturers. Equally important to obtaining the legal authority to independently run its operations was the work MCAZ undertook to develop the infrastructure and processes required to operationalize this new process: establishing the necessary financial procedures, developing standard operating procedures, and hiring and training an internal finance, accounting, and operations team.* *Because MCAZ are funded through generated fees, senior leadership also quickly recognized that a risk-based approach would be the only way to regulate effectively, balancing the need to sustain activities with the criticality of protecting patient safety. An example of this can be seen in how the organization has approached the regulation of medical devices. While MCAZ has had the mandate to regulate medical devices since its inception, the Authority decided to roll out this regulation based on risk, which dictated an initial focus on male condoms in 2005 and medical gloves in 2006 because of the prevalence of HIV/AIDS at the time. With the recent publication of WHO’s model guidelines on the regulation of medical devices* [29]*, MCAZ is again displaying its responsiveness by starting to regulate the import and export of additional medical devices as part of an initial risk analysis.*	

### Utilize risk-based approaches for resource allocation

NRA financial and human resources should not be divided equally among all regulated products and regulatory processes [11, 13]. In LMICs, where available resources are rarely close to commensurate with the needs and expectations of the NRA, adoption of a risk-based approach for resource allocation is crucial [11, 30]. A risk-based approach seeks to channel available resources to regulatory functions and activities that are most likely to facilitate access to quality-assured products and identify and address high-risk quality problems (e.g., a falsified anti-tuberculosis medicine), thereby maximizing the impact of regulatory investments [30, 31]. Generally, such activities include oversight of country clinical trials and manufacturing, country post-marketing pharmacovigilance and quality surveillance, and supply chain security. Moreover, these are typically activities that an agency cannot rely on other agencies to implement.

## Perform risk analysis and implement risk management

**Fig. 3:**
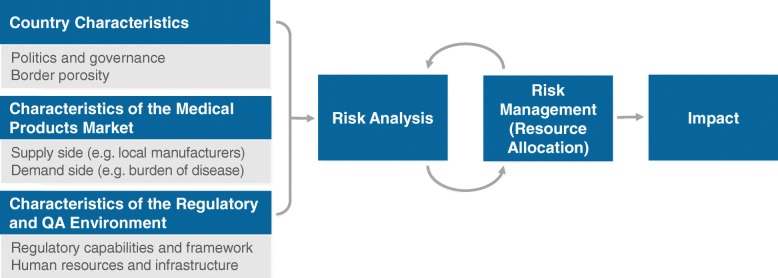
Risk analysis and implementation workflow. Adapted with permission from A Framework for Risk-Based Resource Allocation for Pharmaceutical Quality Assurance for Medicines Regulatory Authorities in Low- and Middle-Income Countries [30]

Investment and technical assistance are needed to support NRAs in performing an initial risk analysis and subsequently implementing risk management activities. A risk analysis uses quantitative and qualitative methods to describe the risks in a system. It includes analyzing the pharmaceutical market, specific country characteristics and the regulatory environment, and it seeks to ascertain attributes that increase (risk triggers) or decrease (risk-mitigating factors) the probability of risk occurrence (Fig. 3) [30, 32]. Risk estimation then calculates the probability and magnitude of each identified risk and ranks them commensurately with available resources. Because of required data needs and environmental familiarity, the best analyses are those that involve all relevant stakeholders, encourage dialogue, and proactively seek to identify critical product quality issues [30, 33]. The results of a risk analysis can then inform risk management activities such as the actual allocation of resources (e.g., financial, human, infrastructural) to regulatory functions based on their risk ranking [30]. This can include, for example, the reallocation of staff from pre-marketing to post-marketing quality surveillance and pharmacovigilance functions.

## Develop systems to monitor and evaluate the impact of risk-based approaches for resource allocation

The final phase of investment needs to be directed towards the development of systems that monitor and evaluate the impact of risk-based approaches for RSS [30, 31]. Impact metrics can include health outcomes, costs and cost savings, efficiency, and sustainability. Risk-based approaches need to be agile, adaptable, and responsive. They should evolve based on the acquisition of new data (e.g., medical product safety surveillance data) and situational changes (e.g., disease outbreaks, conflict). Therefore, ongoing monitoring and evaluation of the allocation of resources based on risk needs to be institutionalized to identify and respond to these catalytic factors and measure the effectiveness of the approach itself. Regulators need to understand and develop the skills required to design and implement monitoring and evaluation systems, including impact evaluation programs to gather and utilize data to inform decision-making, prompt regulatory action, and achieve maximum public health impact.

### Timely access to new quality-assured medical products

#### Strengthen registration efficiency and timeliness

Registration/marketing authorization is the procedure whereby an NRA independently assesses the safety, efficacy, and quality of a medical product and then gives permission for legal marketing in a given jurisdiction [34]. Many LMICs will facilitate myriad drug and vaccine introductions in the coming years [35]. New medical products, including biologics, similar biotherapeutic products (SBPs), medical devices, and vaccines offer health benefits for patients [6]. However, many factors, including the growing number of products seeking registration, their manufacturing complexity, limited evaluation expertise, and the specific regulatory systems required for these products result in an extended backlog for product introduction in many LMICs [6, 19, 36].

## Establish and refine value-added registration processes, resources, and systems

To regulate new products and help ensure their accessibility, the existence of a value-added, product-class specific registration process is a critical and necessary step. These processes must be science-based, transparent, accountable, and predictable. Registration requirements for medical devices, for example, differ from those of other medical products partly because their scope, risk, and complexity vary greatly. A 2015 study in Africa reported that the majority of countries assessed did not have a registration procedure in place for in vitro diagnostics (IVDs) [37]. Registration procedures for devices should therefore follow the guidance of the International Medical Device Regulators Forum (IMDRF) and WHO, both of which recommend that registration of a new medical device be based on a four-tiered risk classification scheme [29, 38]. To support this scheme, existing regional and international resources and systems need to be proactively adapted and implemented in LMICs. An example of this, would be to continue to evolve the WHO Prequalification (PQ) Programme to include new classes of medicines, such as biologics (which has been initiated), medical devices beyond IVDs, and other essential medicines [7]. Donors should also invest in further strengthening functional technical regional harmonization initiatives (RHIs). Once NRAs and RHIs have demonstrated a sufficient and sustainable maturity level (level 3 and 4 in Fig. 2), WHO should recognize their assessment for the purpose of prequalification, as it does now with several mature regulatory authorities. Complementing these efforts is the need to develop and adopt/adapt regulations; related data standards; and functional, fit-for-purpose, robust information management systems (IMS) that address and support the specific manufacturing quality needs of many of these highly complex products, such as SBPs [19, 39].

## Build value-added technical capacity of assessors

Registration of new products requires a cadre of trained and competent assessors, who evaluate registration packages and have knowledge of emerging and existing products, knowledge many LMICs lack [11]. Resources such as existing training platforms, best practices, and procedures, need to continue to be made available to facilitate value-added capacity building for assessors by experienced institutions and networks as discussed in recommendation 2 [7, 17, 40]. It is critical to determine what capabilities are needed at the LMIC level, recognizing that one size does not fit all, to ensure that assessors can review manufacturing dossiers for new products. If products are locally manufactured, the ability to oversee manufacturing in an NRA’s own jurisdiction is a necessary competence. Conversely, if products are imported after having been inspected by a mature NRA or the PQ programme, regulating through reliance on inspection reports and assessments is needed. Likewise, if products are going to be assessed through a regional network or a work-sharing mechanism, the capacity to regulate through and in networks is a skill set that needs to be developed. Building such competencies through a coordinated, specific, goal-focused initiative, such as WHO’s CIP, is highly encouraged.

### Strengthen inspection capacity and effectiveness

The inspection of facilities involved in product development and along the supply chain is integral to assure the quality of medical products. Inspections conducted in accordance with international standards, norms, and guidelines reveal weaknesses and deficiencies as well as actual or predictable errors in production, quality control, storage, or distribution [41]. The complexity and number of new and generic medical products entering LMICs results in increased pressure on already strained local inspectorates that need to identify staff with the relevant expertise to decide when such inspections are needed and whether physical or desk-based (reliance) inspections are most appropriate [7].

## Enhance information sharing and use and reliance on existing inspection resources

To strengthen inspection capacity, information sharing (such as the ability to share full inspection reports among agencies) needs to continue to improve and existing resources need to be utilized effectively. Using these resources and guidelines, such as those published by the Pharmaceutical Inspection Cooperation Scheme (PIC/S), can prevent duplication of efforts and conserve resources, provided local inspectors have access to unredated inspection reports, and the skills to interpret the information therein. Mature authorities should share complete (i.e., unredacted) inspection reports, strive for mutual recognition of inspections, and promote work sharing and more coordination [11, 22, 42]. Mutual recognition of inspection reports was formalized between the U.S. FDA and European Medicines Agency (EMA) at the end of 2017, although this has been standard practice between EMA and Australia, Canada, Japan, Switzerland, and others for a while [43]. Furthermore, the IMDRF has successfully piloted a Medical Device Single Audit Program, which will promote reliance and reduce work load for inspection of medical devices [44]. Support is therefore needed to continue the development and adaption of processes required for LMICs to embed reliance (reduction) and recognition (replacement) [13], including through the sharing of manufacturing inspection information, into their own regulatory processes. This will enable more prudent and risk-based allocation of scarce resources to perform physical inspections of those facilities that have not been inspected and that manufacturer’s use for LMIC markets.

## Build capacity of multi-disciplinary teams of inspectors

Support is needed to build and train multi-disciplinary teams of inspectors with relevant expertise to inspect facilities in local jurisdictions. Training topics should include: assurance of good distribution practices locally, reviewing of external public and unredacted inspection reports, and risk-based post-manufacturing inspection of facilities in the product supply chain. Inspectorates in regions with limited capacity and significant local manufacturing should receive support to obtain PIC/S membership; specifically, this could be for a member state from each of Africa’s Regional Economic Communities where, apart from South Africa, there are no PIC/S participating authorities (Table 4). Investment is also needed to develop internal training plans and programs to ensure that existing staff are continuously learning to meet the challenges of a changing environment and that new staff acquire the knowledge and skills needed to perform their functions. Training implementation should utilize existing platforms, resources, and tools, such as the PIC/S Inspectorates’ Academy, WHO PQ training or secondment opportunities and joint inspections [45].

**Table 4:** South Africa – The road to Pharmaceutical Inspection Cooperation / Scheme (PIC/S) membership

*The South African Health Products Regulatory Authority (SAHPRA), formerly the Medicines Control Council, was invited to join PIC/S in July 2007 – a first for an African country. PIC/S support to NRAs focuses on strengthening their inspectorates by harmonizing good manufacturing practices (GMP) standards and processes for inspection of quality systems, promoting networks and information exchange with other NRAs, and supporting training and capacity building of GMP inspectors. South Africa’s process for PIC/S membership began with application in 1997.* *A key step in enabling this process was ensuring buy-in and support of all stakeholders, including government and industry. Legislative amendments to the Medicines Act were passed in 2003 to further strengthen licensing arrangements for the manufacturing, import, export, wholesaling, and distribution of medicines. During the application process, the NRA reviewed its existing procedures to identify areas of improvement. The NRA designed and implemented a quality management system that incorporated a quality manual, technical guidelines, and standard operating procedures for all inspection activities, highlighting confidentiality, code of conduct, ethics, and conflict of interest. Improving the capacity of the inspectorate also focused on strengthening administrative, structural, and technical components. Through a series of workshops held between 2004 and 2006, local industry was engaged to adopt the new PIC/S GMP guidelines and ensure their effective implementation, compliance, and enforcement. This process was supported by local technical experts as well as experts from PIC/S member countries to ensure a common interpretation and understanding of the technical principles by both inspectors and industry. Removing barriers to effective communication and encouraging transparency and feedback were key to ensuring the support and compliance of industry stakeholders.* *In September 2006, PIC/S inspectors assessed the NRA’s progress and made several observations, which were addressed, resulting in admission the following year as PIC/S′ 31st Participating Authority. SAHPRA continues to strengthen its regulatory capacity by tapping into the pool of PIC/S expertise. PIC/S membership has provided opportunities for networking with counterpart NRAs; promoting quality systems and participating in Joint Visit Programmes, Expert Circles, subcommittees, and working groups.*	

### Limited evidence-based data to support post-marketing regulatory action

#### Develop and implement risk-based post-marketing quality surveillance systems

Post-marketing quality surveillance (PMQS) is the process of routinely sampling and testing medical products in a planned, value-added schema, following their market approval. As a regulatory function, risk-based PMQS complements traditional pharmacovigilance activities, generating data on product quality to identify supply chain vulnerabilities and highlight product quality issues in the local market (see Table 5). However, current regulatory systems in many LMICs are not equipped to develop and implement effective and sustainable PMQS systems and consequently cannot generate and utilize the data required to support needed regulatory actions [6, 11, 21]. This is due, in some situations, to limited recognition of the value of PMQS as a critical regulatory function, and a lack of practical, tailored guidance and tools [11, 46].

**Table 5:** Therapeutic Goods Administration – Risk-based post-marketing quality surveillance as a critical regulatory function

*Australia’s NRA, the Therapeutic Goods Administration (TGA), has been 100% cost recovered (autonomous, but self-funded) since the late 1990s. Cost-recovery compels the organization to think strategically about the implementation of its regulatory functions. In 2011, TGA assessed its testing program, identified areas of improvement, and set a roadmap for investing in and establishing a risk-based PMQS program for its programmed testing activities. Risk management standards were consulted and adapted to TGA’s context, principles were identified (e.g., the program would need to be dynamic, iterative, and responsive to change), and a framework and related foundational processes were developed (e.g., risk analysis, risk management, impact evaluation, risk communication).* *The program took 3 years to develop, primarily because of the complexity of designing a system that would cater to and enable risk scoring of all product categories (e.g. over-the-counter medicines, complementary medicines, and medical devices). The program has now been active for several years and, importantly, publishes testing data through TGA’s website. Products that undergo a more intensive pre-market assessment (e.g. prescription medicines), tend to score lower and are therefore tested less frequently. However, groups within categories can be scored differently. For example, both vaccines and biological medicines are regulated as prescription medicines in Australia but score more highly than other prescription medicines in general. This is due to the increased complexity of the products and increased complexity of manufacturing; in the case of vaccines, it is also because they are given to an entire cohort of healthy children every year. The risk category of a product can also be elevated (a dynamic program) in response to specific factors, such as poor GMP, failed laboratory testing results, or adverse event reports.* *Key to the success of the program was political will and staff commitment, which enabled the development and implementation processes to be followed thoughtfully and have led to less resource waste, increased effectiveness (e.g., shifting the type of products that are tested), and identification of poor-quality products.*	

## Establish recognition of the value of risk-based post-marketing quality surveillance throughout the supply chain

PMQS is often undervalued as a regulatory function, particularly in LMICs where limited resources cannot be deployed equally across all functions and are typically allocated to pre-market functions, such as registration [46]. For example, while the AU Model Law indicates that an NRA “may” institute PMQS, it specifies that a national pharmacovigilance program “shall” be established [28]. Local NRAs therefore need support to engender recognition of the value of developing and implementing PMQS systems. Pre-market activities cannot identify local supply chain disruptions, cold chain excursions, illicit trafficking, or poor distribution practices that lead to product degradation. While pharmacovigilance can be a valuable identifier of quality problems with attendant patient harm, its focus is principally on adverse events, such as adverse drug reactions. PMQS is one of the most patient-centered regulatory functions, monitoring product quality issues immediately prior to their use by patients. As such, its implementation is as central as registration, inspection, or pharmacovigilance.

## Develop and implement risk-based post-marketing quality surveillance programs, supporting tools, and communication strategies

Once recognition of the value of PMQS is established, investment needs to focus on developing programs using well-established guidelines, tools, and best practices, tailored to country contexts [46, 47]. Passive systems for reporting of suspected quality issues should also be established. The program design needs to take into account the local pharmaceutical sector, scope of sampling, governance and transparency, laboratory and personnel capacity development requirements, coordination, communication, financing, and sustainability [46]. Complementing the design of PMQS programs is the need to continue to examine available and emerging technologies that can be used for advanced analytical screening [4, 17, 48, 49]. Investment is then needed to support the implementation of PMQS activities in LMICs, notably sampling following a validated methodology, testing in internationally accredited laboratories, and the subsequent appropriate dissemination, communication, and use of data to support appropriate regulatory action. The program design should also identify key stakeholders and their roles and responsibilities, and outline targeted advocacy and training priorities, such as strengthening of national quality control laboratories. Beyond training, support is needed for the development of local and regional databases that complement WHO’s Global Surveillance and Monitoring System [50]. Data standards that define how quality control data are collected and stored are urgently needed as are procedures that identify the practical aspects of communicating results, sharing information, and initiating enforcement action.

### Strengthen regulatory management of manufacturing variations

#### Develop and implement risk-based programs to incorporate post-marketing manufacturing variations into marketing authorizations

During the lifecycle of an approved medical product, changes to the authorized manufacturing process are inevitable. Whether to improve manufacturing efficiency, utilize new manufacturing technologies, move physical locations of manufacturing facilities, or change suppliers of manufacturing components, assessing and authorizing these variations to the marketing authorization for the product require regulatory agency focus, resources, and specific knowledge [51]. In many LMICs, these resources and knowledge are often not available. Because of this, manufacturing variations are often backlogged, resulting in shortages when a product manufactured in compliance with the still current authorization in a country is no longer available. Investment in the development of local processes and systems that rely on the approval of variations by the WHO PQ Programme or mature NRAs could have a significant impact on continued quality product availability, provided the variation authorized was for the specific product that is being shipped to the country.

## Conclusion

The globalization of medical product manufacturing means no single regulatory authority alone can guarantee the safety of all products in its country’s market [9, 11]. In today’s linked supply chains, medical product quality and safety in one country increasingly depends on systems in other countries. Strengthening medical product regulatory systems in LMICs in value-added ways enables reliance and work sharing and fosters a coordinated approach as a part of the drive for universal access to quality healthcare [6, 42]. The recommendations identified herein, if implemented, can increase timely access to quality-assured medical products and improve detection of and response to SF medical products. The recommendations focus on advancing reliance and regulatory networking efforts, institutionalizing sustainability, utilizing risk-based approaches, enhancing value-added registration and inspection capacity, and developing systems for the generation of data to support post-marketing regulatory action. Our intent is for this paper to be used as a blueprint to help guide the next wave of investment in RSS in order to realize the aspirational but universally agreed-upon Sustainable Development Goal of good health and wellbeing for all [52].

## Abbreviations

APEC: Asia-Pacific Economic Cooperation
AU: African Union
CIP: Coalition of Interested Partners
EMA: European Medicines Agency
FDA: Food and Drug Administration
GBT: Global benchmarking tool
GMP: Good manufacturing practices
IMDRF: International Medical Device Regulators Forum
IVD: In vitro diagnostics
LMICs: Low- and middle-income countries
MCAZ: Medicines Control Authority Zimbabwe
NRA: National Regulatory Authority
PIC/S: Pharmaceutical inspection cooperation / scheme
PMQS: Post-marketing quality surveillance
PQ: Prequalification
RHI: Regional harmonization initiative
RSS: Regulatory system strengthening
SAHPRA: South African Health Products Regulatory Authority
SBP: Similar biotherapeutic product
SF: Substandard and falsified
TGA: Therapeutic goods administration
WHO: World Health Organization

## Footnotes

1 For the purposes of this article, the definition of access encompasses physical accessibility (availability within reasonable reach of those who need them), financial affordability (ability to pay without financial hardship), and acceptability (willingness to seek medical products when needed) [1].

2 For the purposes of this article, the definition of medical products includes medicines, biologics, vaccines, and medical devices.
